# Local Mercurial Fumigation

**Published:** 1882-02

**Authors:** 


### LOCAL MERCURIAL FUMIGATION.

The subject of local mercurial fumigation has lately been attracting some attention in Great Britain. Dr. F. H. Kane (*Dublin Journ. of Med. Science*, Nov. 1874) described and figured a simple apparatus by which the vapor of sublimed calomel could be readily and with precision directed on any desired region, such as the nose, mouth, pharynx, or skin. In 1872 (*Brit. Med. Journal*) surgeon Moffitt described a very similar apparatus, and renewed attention was directed to the subject by a correspondence in the same publication in 1880 (vol. ii). Moffit volatilized from three to five grains of calomel, and directed it by means of a stream of air as in the spray-producers. Quite recently, Dr. Walter G. Smith read a paper on the results obtained by him since the publication of Kane's paper. He has used it frequently, and claims for it exceptionally gratifying and rapid curative results in similar cases to those in which iodoform is applicable, that it is painless even when directed on most sensitive surfaces, that it is easy of application, and capable of precision in use, and that it does not salivate.—*Brit. Med. Journal*, May 7, 1881.

